# Structural analysis of leucine, lysine and tryptophan mitochondrial tRNA of nesting turtles *Caretta caretta* (Testudines: Chelonioidea) in the Colombian Caribbean

**DOI:** 10.7717/peerj.9204

**Published:** 2020-06-18

**Authors:** Harvey Infante-Rojas, Leonardo Marino-Ramirez, Javier Hernández-Fernández

**Affiliations:** 1 Department of Natural and Environmental Sciences, Genetics, Molecular Biology and Bioinformatics Lab, Jorge Tadeo Lozano University, Bogotá, Cundinamarca, Colombia; 2 NCBI, NLM, NIH Computational Biology Branch, Bethesda, MD, USA

**Keywords:** tRNA mitochondrial, *Caretta caretta*, Bioinformatics, Canonical structure, Structural biology, 2D structure, 3D structure

## Abstract

The understanding of the functional properties of mitochondrial transfer RNA (mt tRNAs) depend on the knowledge of its structure. tRNA acts as an interface between polynucleotides and polypeptides thus, they are key molecules in protein biosynthesis. The tRNA molecule has a functional design and, given its importance in the translation of mitochondrial genes, it is plausible that modifications of the structure can affect the synthesis of proteins and the functional properties of the mitochondria. In a previous work, the mitochondrial genome of an individual of the nesting *Caretta caretta* of the Colombian Caribbean was obtained, where specific mutations were identified in the only tRNA^Leu (CUN)^, tRNA^Trp^ and tRNA^Lys^ genes. In order to analyze the effect of these mutations on these three mt tRNAs, the prediction of 2D and 3D structures was performed. Genes were sequenced in 11 nesting loggerhead turtles from the Colombian Caribbean. Two-dimensional structures were inferred using the ARWEN program, and three-dimensional structures were obtained with the RNA Composer 3D program. Two polymorphisms were identified in tRNA^Trp^ and another one was located in tRNA^Lys^, both specific to *C. caretta*. The thymine substitution in nucleotide position 14 of tRNA^Trp^ could constitute an endemic polymorphism of the nesting colony of the Colombian Caribbean. Two 2D and three 3D patterns were obtained for tRNA^Trp^. In the case of tRNA^Lys^ and tRNA^Leu^ 2D and 3D structures were obtained respectively, which showed compliance to canonical structures, with 4 bp in the D-arm, 4–5 bp in the T-arm, and 5 bp in the anticodon arm. Moderate deviations were found, such as a change in the number of nucleotides, elongation in loops or stems and non-Watson–Crick base pairing: adenine–adenine in stem D of tRNA^Trp^, uracil–uracil and adenine–cytosine in the acceptor arm of the tRNA^Lys^ and cytosine–cytosine in the anticodon stem of the tRNA^Leu^. In addition, distortions or lack of typical interactions in 3D structures gave them unique characteristics. According to the size of the variable region (4–5 nt), the three analyzed tRNAs belong to class I. The interactions in the three studied tRNAs occur mainly between D loop—variable region, and between spacer bases—variable region, which classifies them as tRNA of typology II. The polymorphisms and structural changes described can, apparently, be post-transcriptionally stabilized. It will be crucial to perform studies at the population and functional levels to elucidate the synthetic pathways affected by these genes. This article analyses for the first time the 1D, 2D and 3D structures of the mitochondrial tRNA^Lys^, tRNA^Trp^ and tRNA^Leu^ in the loggerhead turtle.

## Introduction

The loggerhead turtle *Caretta caretta* (Linnaeus, 1758*)*, lives in tropical and warm oceans (Pritchard & Mortimer, 1999). It is an important component of complex ecological marine and coastal systems (Eckert et al., 1999; Buitrago, 2003; Pritchard, 2004). It favors the resilience of marine environments maintaining the balance in ecosystems and food chains they occupy through the control of mollusks, crustaceans and other marine invertebrate populations (Machado & Bermejo, 2012). Current research for the management and conservation of sea turtles, in which molecular studies and DNA mitochondrial analysis (DNAmt) are the baseline, are being developed (Stewart & Dutton, 2012; Duchene et al., 2012). Mitogenomes of sea turtles have been completely sequenced (Kumazawa & Nishida, 1999; Duchene et al., 2012; Drosopoulou et al., 2012; Otálora & Hernández-Fernández, 2018; Hernández-Fernández & Delgado Cano, 2018) and the efforts have been directed towards the use of mitochondrial genes as molecular markers, allowing to explain phylogeny (Shanker et al., 2004; Shamblin et al., 2012), the evolution, the migration routes (Zardoya & Meyer, 1998; Chiari et al., 2012), the population structure and dispersion centers (Hillis, Richardson & Richardson, 1995; Bolten et al., 1998), as well as the identification of polymorphism and haplotypes (Carreras et al., 2011). Otálora & Hernández-Fernández (2018), who obtained the mitochondrial genome from a nesting *C. caretta* individual of the Colombian Caribbean, identified point mutations in the RNA mitochondrial transfer, tRNA^Leu(CUN)^ tRNA^Trp^ and tRNA^Lys^ that could modify their tertiary structure, which could eventually compromise the synthesis of peptides. Point mutations in genes that encode mitochondrial RNAs can generate in these, non-canonical secondary structures and unconventional folds in their tertiary structure (Laslett & Canback, 2008). To fulfill their biological function, tRNAs have very specific structural properties that allow recognition and interaction with various partners, such as aminoacyl-tRNA-related synthetases and the ribosome (Helm et al., 2000). Most tRNAs fold into the “canonical” cloverleaf secondary structure, and then into a tertiary structure known as L-shaped (RajBhandary & Soll, 1995). The secondary and tertiary structures of tRNA molecules are well conserved in almost all organisms (Widmann et al., 2010) so it has been possible to establish conserved regions and invariant residues, or discriminating elements that confer specificity for the recognition of amino acids (Alexander, Eargle & Luthey-Schulten, 2010). However, mitochondrial tRNAs (mt tRNAs) from metazoans often move away from classical structures (Frazer & Hagerman, 2008). Under certain circumstances, point mutations in mitochondrial tRNA genes lead to structurally invalid tRNAs (Helm et al., 2000), and may even change specificity after a single mutation in the anticodon (Widmann et al., 2010). This problem has been studied primarily in human mt tRNA, because mutations can lead to mitochondriopathies (Pütz et al., 2007; Suzuki, Nagao & Suzuki, 2011). Therefore, the structural analysis of mt tRNAs of *C. caretta* is important to understand the way in which their folding occurs, identify conserved regions, and for the definition of identity elements.

Despite the importance of tRNA mt and the detailed studies that have been conducted in humans, in sea turtles there is currently no information that describes or analyzes these important molecules. The availability of large databases that contain thousands of tRNA sequences from hundreds of complete genomes, have promoted the development of a new field of “tRNAomics” (Marck & Grosjean, 2002), nevertheless, the secondary and tertiary structures of the majority of tRNA are currently unknown. This situation has given rise to a high demand in structural biology to infer the secondary and tertiary RNA structures using prediction methods (Popenda et al., 2012). The objective of this study was to describe and analyze the primary, secondary and tertiary structures of mitochondrial tRNA^Leu^, tRNA^Trp^ and tRNA^Lys^ of *C. caretta*, in a group of nesting individuals of the Colombian Caribbean, to identify mutations, describe structural modifications and its possible implications in the functionality of these molecules. Due to restrictions in carrying out the sampling, given that *C. caretta* is a vulnerable species, only 11 blood samples were obtained; therefore, the scope of the study is moderate. Nevertheless, it represents a baseline for structural and functional studies of mitochondrial tRNA of the loggerhead turtle at the population level that may be used for its conservation. This research represents the first analytical exploration of tRNAs in the *C. caretta* turtle.

## Materials and Methods

### Biological samples

Peripheral blood samples were collected from 11 healthy captive loggerhead turtle of undetermined sex and maintained at ambient temperature (average 30 °C, minimum 27 °C) in an outdoor seawater pool at the CEINER Oceanarium in San Martin de Pajares island, Cartagena, and two from Don Diego beach (11°16′N y 73°45′O) in PNN Tayrona—Santa Marta characterized by a semi-arid climate (identified in this study as CC1, CC2, CC3, CC4, CC5, CSM-1, CSM-2, CSM-3, CR-2, 2C y 3C according to molecular biology laboratory nomenclature). The blood was obtained from the dorsal cervical sinus in accordance with Dutton (1996). The samples were placed in sterilized tubes with Tris-EDTA buffer 0.1 M (GreinerBio-one^®^, Kremsmünster, Austria) solution and were transported at 4 °C to the Molecular Biology lab of the Universidad Jorge Tadeo Lozano, Bogota campus. The samples were collected following the ethical standards established by the legislation and the study obtained permission from the Ministry of Environment and Territorial Development (No 24 of June 22, 2012) and the Genetic Resources Access contract (No 64 of April 2013)

### Extraction of DNA from the blood tissue

The genomic DNA was obtained from eleven blood samples using the GF1GF-1 Tissue DNA kit (VIVANTIS-Malasia). The DNA obtained was visualized by electrophoresis in agarose gel at 1% p/v with Safeview stain (two µg/ml) (Fig. S1). The DNA was quantified using the Nanodrop 1000 Spectrophotometer equipment and was analyzed with the ND-1000 V3.7.1, (ThermoScientific, Maltham, MA, USA) program.

### PCR amplification and sequencing

The selection of 3 of the 22 tRNA (tRNA^Trp^, tRNA^Lys^ and tRNA^Leu^) was sustained in the prior results obtained by Otálora & Hernández-Fernández (2018) who identified point mutations in these three genes. For the PCR amplification of the tRNA genes, three primer pairs were used; cc7, cc11 and cc16 (Beltrán et al., 2013), that amplified three fragments of 800 bp that contain the coding sequences of tRNA^Trp^, tRNA^Lys^ and tRNA^Leu^ respectively (Fig. S2). The mixture of PCR reaction contained 1 unit of MyTaq DNA Polymerases (high reliability) (Bioline Inc., California, USA, EE.UU.), 2 mM of MgCl2, 1 mM of primer, 1X PCR tampon (50 mM of KCl and 10 mM of Tris-HCl pH 8,3), and 0,2 mM of deoxyribonucleotide (DNTP´s), in a final volume of 25 µL. The thermocycling program consisted in one step of initial denaturing of five minutes at 95 °C, followed by 35 cycles of 95 °C for 1 min, 40 and 44 °C (depending on the primer) for one minute and 72 °C for another minute, with a final extension of 10 min at 72 °C. The PCR was conducted with an automatic thermocycler (Labocon Systems Ltd, Hampshire, UK). The PCR products were purified using the GF-1 Gel DNA Recovery kit (Vivantis, MALASIA) and were sequenced in both directions (5′–3′ and 3′–5′) using the tagDyeDeoxy Terminator Cycle-sequencing method, in an 3730XL sequencer (Applied Biosystems, Foster City, CA, USA) at SSIGMOL (Universidad Nacional de Colombia: http://www.ssigmol.unal.edu.co/).

### Data analysis

The tRNA sequences were assembled using the Geneious R6^®^ program (Biomatters, Ltd., New Zealand). With the online tool BLAST (Altschul et al., 1990) (http://blast.ncbi.nlm.nih.gov/) basic local alignment was conducted to determine the similarity percentage of the sequences obtained with the sequences of *C. caretta* previously described. All sequences obtained in this study and the sequences coded for the same tRNA in the seven sea turtle species previously reported in GenBank were aligned using the algorithm ClustalW (Thompson, Higgins & Gibson, 1994). The inference of the 2D structures of the tRNA was conducted with the software ARWEN (Laslett & Canback, 2008) (http://130.235.46.10/ARWEN/). Parameters: default metazoan mitochondrial code. Output structures were manually curated. The prediction of the 3D structures was conducted using the online program RNA composer 3D (Popenda et al., 2012) (http://rnacomposer.cs.put.poznan.pl/). Modeling was performed with ‘batch mode’ with secondary structures as the input, and was visualized through the program Geneious R6^®^ (pdb format), were the distance between the anticodon and unpaired nucleotide in the 3′ end of each structure was measured. The potential interaction networks and the non-canonical base pairs were identified for the tertiary structures obtained. The nucleotide sequences and inferred structures were identified with the same name of the individual to which they belonged. The positions of the nucleotides were numbered according to the conventional standards (Sprinzl et al., 1998), and definition of canonical tRNA cloverleaf and numbering of nucleotides (Salinas-Giegé, Giegé & Giegé, 2015).

## Results

### DNA extraction, amplification and sequencing

The genomic DNA obtained from the blood sample of 11 individuals of (*C. caretta*) loggerhead turtle were of good quality, obtaining concentrations of ±50 ng/µL and purity in a range of 1.8–2.0. Lastly, 11 sequences of the tRNA^Trp^ and tRNA^Leu(CUN)^ mitochondrial genes respectively and nine sequences for the tRNA^Lys^ genes were obtained; two of the sequences did not present clear chromatograms, therefore, they were not taken into account. On some occasions there may be problems in the sequencing reaction that prevent having a good sequence, related to: The primer could be linked in several positions to the template DNA, it is also possible that more than two PCR products have been amplified and sequenced in parallel presenting wrong chromatograms, in addition the primers of the original PCR may not have been removed. These sequences obtained were deposited in the GenBank database (accession numbers KX063643–KX063673). The BLAST analysis established a similarity percentage of 98–100% between the sequences obtained and the mt-tRNA sequences deposited in GenBank.

### Multiple alignment of sequences

The molecular analysis of tRNA^Trp^ was conducted using 78 nucleotide positions, of which, 62 positions were identical for the seven species, with a conservation of 79.4% of this gene (Fig. S3). *C. caretta* was differentiated from the other taxa because the tRNA^Trp^ gene revealed an additional thymine in position 26 and a C→T transition in position 48 that represent interspecific variations. A mutation in position 14 (transition C→T) was identified in this same gene, shared by nine of the eleven turtles of this study, but absent in the previously described sequences. This result is in agreement with the one described by Otálora & Hernandez (2015), Otálora & Hernández-Fernández (2018). Apparently, this mutation would be fixed in the nesting colony of the Colombian Caribbean, constituting an endemic haplotype. This hypothesis is supported by the genetic differences identified in populations of different geographic areas (Bowen, Nelson & Avise, 1993; Encalada et al., 1996) as a result of reproductive isolation and low genetic flow that could cause the philopatry (Cuadros-Arasa, 2013).

The analysis of the tRNA^Lys^ gene sequences was based in 73 nucleotide positions, of which, 66 were identical for all the species (90.4% of conservation for this gene). Seven mutations between positions 52 and 62 were identified (Fig. S4). *C. caretta* was uniform in its sequences, with a C→T transition in position 62 that was not shared with any other species. Only the Colombian sequence described by Otálora & Hernandez (2015) presented a transversion in the nucleotide position 18 (mutation at the level of the individual).

The tRNA^Leu^ gene presented 72 nucleotide positions in 66 identical places in all the sequences (98.1% of conservation). Six mutations were identified, four of which were contributed by *D. coreacea*, evidencing the divergence of this species (Eckert et al., 1999). The majority of sequences of *C. caretta* presented an adenine at position 43 as described by Otálora & Hernández-Fernández (2018); nevertheless, two of the sequences obtained in this study and two of the sequences previously described presented a A→G transition in the same position (Fig. S5).

### Secondary structures

**Figure 1:**
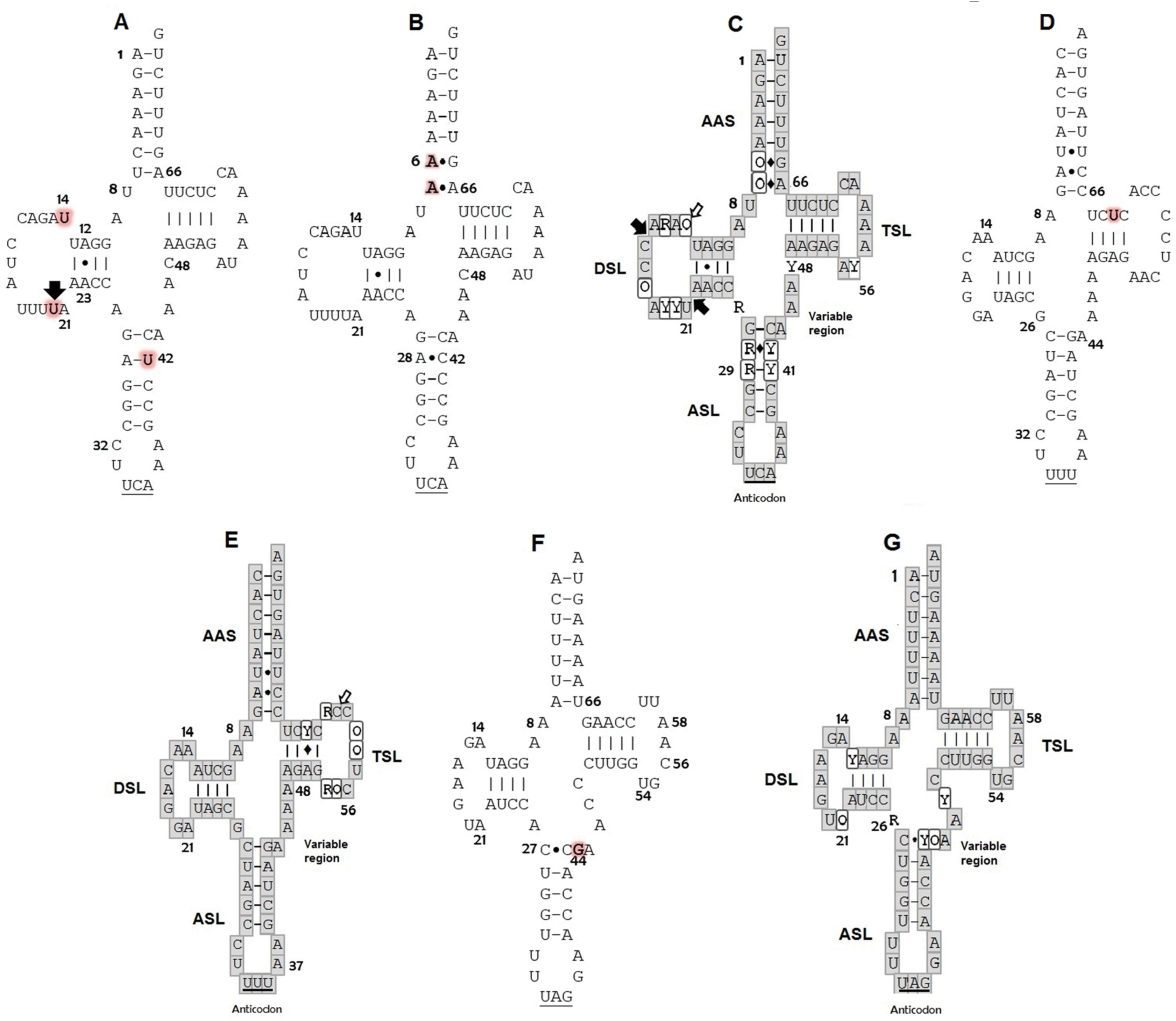
Putative secondary structures: (A) tRNA^Trp^ of *C. caretta* dominant motif; (B) tRNA^Trp^ of *C. caretta* motif obtained for the individual CC2; (C) tRNA^Trp^ consensus; (D) tRNA^Lys^ of *C. caretta*; (E) tRNA^Lys^ consensus; (F) tRNA^Leu(CUN)^ of *C. caretta*; (G) tRNA^Leu(CUN)^ consensus. The letters highlighted in red indicate distinctive nucleotides or mutations. The numbers indicate the position in the structure. The black arrows indicate insertion and the white arrows indicate deletion. The Watson–Crick pairings are represented with bars and the non-Watson–Crick pairings are indicated with points. The consensus structures gather the motifs and mutations of the seven species of sea turtles found for each tRNA, in which the bases in the gray boxes are invariant, the Y represents semi variant positions with conservation of pyrimidine, the R semi variant position with conservation of purines and the O represents variant positions without a nucleotide family. The diamonds indicate that at that position a canonical or non-canonical pairing can present itself. Subdomains: Amino acid Accepting Stem (AAS), D-Stem Loop (DSL), Anticodon Stem Loop (ASL), T-Stem Loop (TSL). Positions of nucleotides are numbered according to conventional rules (Sprinzl et al., 1998).

The secondary structures inferred from the tRNA^Trp^ sequences presented just one shared motif by ten of the eleven sequences obtained in this study (Fig. 1A). This motif was characterized by having a T-loop composed of 7 nucleotides, anticodon loop with 7 nucleotides and a larger one, D-loop, composed of 13 nucleotides (Table 1). The large size of this loop is because this gene presented a size of 77 bp, 4 more than the 73 bp generally described for the same gene in other species (e. a. the hawksbill turtles, *Eretmochelys imbricata*) (Jung et al., 2006). The D-stem is formed by 4 pairings including one non-canonical pair, adenine–adenine, that could be recognized as a secondary interaction conserved in the tRNA^Trp^ structure, a characteristic presented by the seven species of sea turtles (Fig. 1C). A second 2D structural motif for the tRNA^Trp^ gene was identified from the sequence corresponding to the individual *CC2* (Fig. 1B) that was differentiated by the presence of a non-canonical pairing A_28_–C_42_ in the anticodon stem as a result of the T→C transition identified in the multiple alignment in position 48. In addition two new mutations in a non-complementary A_7_–A_66_ and a non-canonical A_6_–G_67_ pairs in the acceptor stem (because of C→A and T→A transversion in positions 6 and 7) its creating a risk of interrupting the postranscription processes, such as aminoacylation (Blakely et al., 2013). When comparing the motifs obtained for tRNA^Trp^ of *C. caretta* with the consensus structure of tRNA^Trp^ of sea turtles (Fig. 1C), it was observed that the variable region is small, with only 4 nucleotides, and that the D loop contains the majority of variant positions, suggesting that this region presents greater freedom to mutate within the tRNA^Trp^ molecule when remaining conserved in the variable region.

**Table 1:** Characteristics identified in the secondary putative structures of the mitochondrial tRNA^Trp^, tRNA^Lys^ and tRNA^Leu(CUN)^ of the nesting *Caretta caretta* of the Colombian Caribbean.

tRNA	Size (b)	Acceptor stem (pb)	D-arm		T-arm		Anticodon arm		Variable Region (b)	Connecting bases		Non-canonical Pairs (positions)
			Stem (pb)	Loop (b)	Stem (pb)	Loop (b)	Stem (pb)	Loop (b)		Acceptor /D-Stem	D-Stem/ Anticodon Stem	
Lys	73	8	4	7	4	9	5	7	4	2	1	U–U (5A–67)
												A–C (6–66A)
Leu	72	7	4	7	5	7	5	7	5	2	1	C–C (27–43)
Trp	77	7	4	13	5	7	5	7	4	2	1	A–A (12–23)
Trp2^1^	77	7	4	13	5	7	5	7	4	2	1	A–G (6–67)
												A–A (12–23)
												A–C (28–42)
												A–A (7–66)

**Note:**

1 Second tRNA^Trp^ motive identified in an individual from this study.

For the tRNA^Lys^ gene a 2D structural motif was identified (Fig. 1D) in which the T-loop with nine nucleotides is larger than the D with 7 (Table 1). In the D loop, two residues of invariant guanine were identified (G_18_ and G_19_), identical to the canonical tRNA (Westhof & Auffinger, 2001). The distinctive characteristic of this structure is the acceptor stem conformed by 8 intracatenary pairings and not by 7 as presented in the canonical tRNA (Westhof & Auffinger, 2001; Giegé et al., 2012). In addition, two of these pairings are of the non-Watson–Crick type. This characteristic, typical of tRNA^Lys^ in sea turtles, is shared by the other six species (Fig. 1E). On the other hand, at position 62, thymine was observed to characterize the sequences of *C. caretta*. This generates a canonical pairing A_52_–U_62_ in the T-stem (Fig. 1D), absent in the other turtle species.

The tRNA^Leu(CUN)^ analysis produced a secondary structure (Fig. 1F). A mutation at position 44 was identified, which did not generate changes in the secondary structure. In this structure it was observed that the D/T loops and anticodon are formed by 7 bases (Table 1). The variable region presented 5 nucleotides (one more than in the tRNA^Trp^ and tRNA^Lys^). A non-canonical pairing at the first base pair of the acceptor stem was identified, a characteristic shared by the secondary consensus structure of sea turtles.

### Tertiary structure

**Figure 2:**
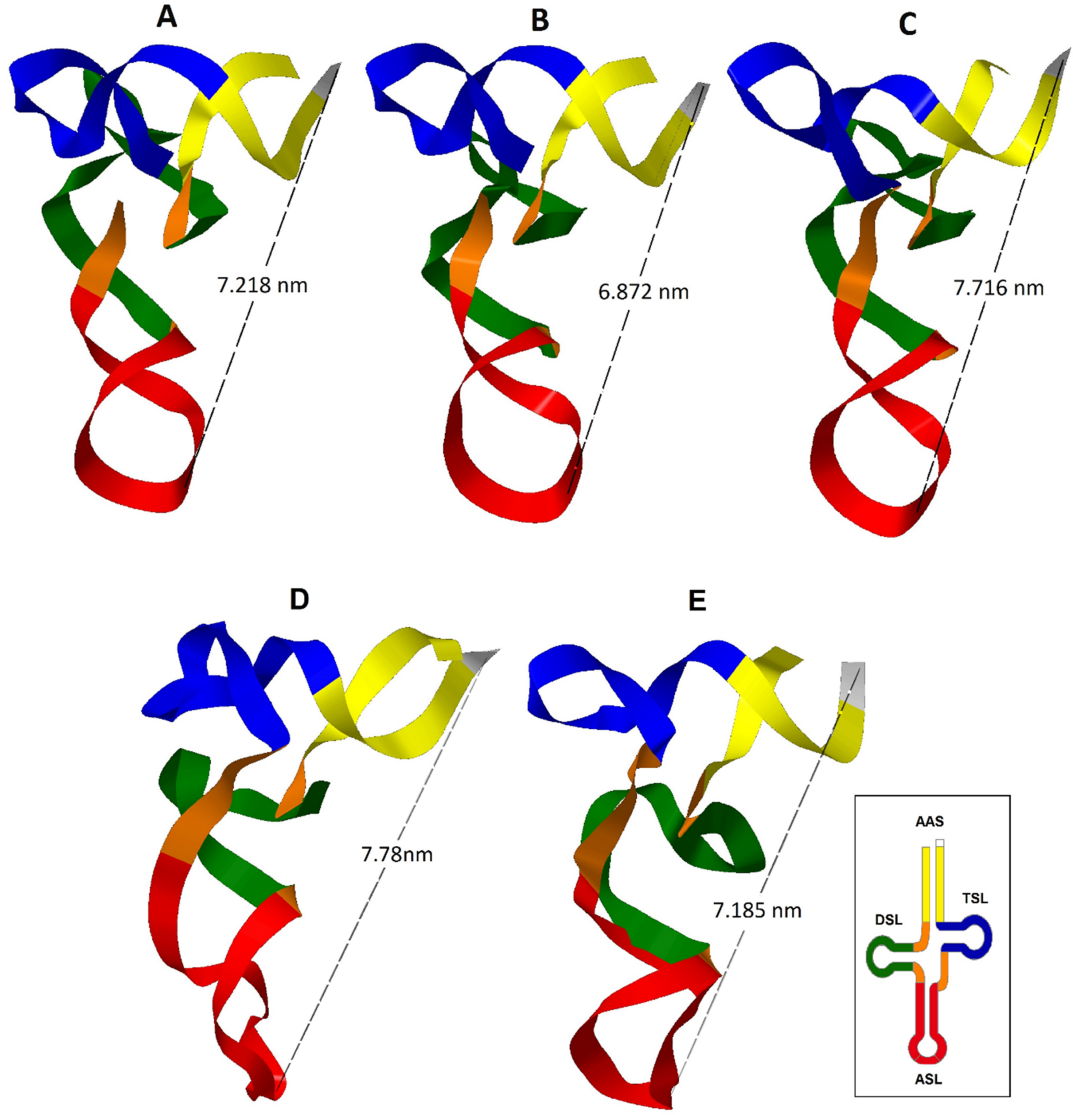
Inferred tertiary structures in the model of strips for the mitochondrial tRNA, tRNA^Trp^, tRNA^Lys^ and tRNA^Leu(CUN)^ for a group of *Caretta caretta* nesting turtles in the Colombian Caribbean. (A) tRNA^Trp^ dominant motif; (B) tRNA^Trp^ motif identified in the individuals CSM-3 and CC5; (C) tRNA^Trp^ motif identified in the individual CC2; (D) tRNA^Lys^; (E) tRNA^Leu(CUN)^.

Three tertiary structures from the eleven sequences analyzed of the tRNA^Trp^ gene were obtained (Fig. 2). The CSM-3 and CC5 individuals presented a tertiary structure that differs from (Fig. 2B) the dominant motif for this gene (Fig. 2A), because they had a cytosine mutation for a thymine at position 14. This transition generated a greater folding of the D-loop producing a larger bend in the nucleus of the molecule, shortening the distance between the anticodon and the 3′ end. This change shows the susceptibility of the tRNA to present distortions in its architecture for a point mutation (Laslett & Canback, 2008).

A tRNA is aminoacylated if it has a distance between the anticodon and the CCA termination at the 3′ end between 7.5–8.0 nm (Westhof & Auffinger, 2001; Suzuki, Nagao & Suzuki, 2011). The predominant tertiary motif of tRNA^Trp^ and the motif found for individuals CSM-3 and CC5 presented a distance of 7.218 and 6.872 nm respectively; at this distance there is still a need to add the length of the CCA sequence, which is added at the tRNA in a postranscriptional manner, a process that could compensate the remaining distance (Giegé et al., 2012). The tertiary motif obtained for the tRNA^Trp^ from the sequence belonging to the individual *CC2* (Fig. 2C) showed a length within the functional range (7.716 nm). This length for this motif is attributed to the presence of the non-canonical pairing and the non-complementary pair identified in the secondary structure that distorted the helix that forms the acceptor stem. According to Fritsch & Westhof (2005) only a small number of non-Watson–Crick base pairs may be incorporated in the stems without interrupting the helical structure, but they anyhow cause distortions that perturb normal function. In this case in which the helix is only 7 base pairs long, the presence of two non-canonical stocked pairs produces this notable distortion, because definitely pathogenic mutations are mainly produced in the stems (Blakely et al., 2013).

From the inferred 2D structure for the tRNA^Lys^, a 3D structure was obtained (Fig. 2D) which showed a close disposition to the typical “L” folding, maintaining opposites in the 3′-end and the anticodon. The T-loop presented a greater torsion than the one commonly presented in a typical tRNA, caused perhaps by the larger size of this loop producing a folding over it. The presence of two non-Watson–Crick pairs evidenced in the 2D structure generated a distortion of the helix of the acceptor stem where the major groove is wider and the minor groove is more closed. In the anticodon loop an atypical distortion was observed that could cause problems in the codon-anticodon alignment; nevertheless, it is known that modified nucleosides are inserted in the anticodon loop and are necessary for its stability (Suzuki, Nagao & Suzuki, 2011). The distance between the anticodon and the 3′-end of 7.78 nm is within the functional range (Westhof & Auffinger, 2001; Suzuki, Nagao & Suzuki, 2011).

The tRNA^Leu(CUN)^ presented a structural motif (Fig. 2E), even when a mutation in the variable region was found. The A→G transition in position 43 did not produce changes in the 3D structure, showing that the variable region is capable of accomodating variations in the number of nucleotides to try to maintain the general architecture of the tertiary structure (Rich & RajBhandary, 1976).

### Tertiary interactions

To obtain the typical 3D structure in “L” form, require tertiary interactions between the loops and the helices (Suzuki, Nagao & Suzuki, 2011). In this study, the network of tertiary interactions canonical was validated (Giegé et al., 2012) for each one of the tRNA analyzed. Table 2 shows the proposed tertiary interactions for mitochondrial tRNA, tRNA^Trp^, tRNA^Lys^ and tRNA^Leu(CUN)^ of *C. caretta*, based on the similarity and coincidence of residues with the interactions described for canonical tRNA from the visual examination of the secondary structures.

**Table 2:** Potential tertiary interactions in the mitochondrial tRNAs, tRNA^Trp^, tRNA^Lys^ and tRNA^Leu(CUN)^ of *Caretta caretta*.

Interaction number	1	2	3	4	5	6	7
Nucleotide positions	15–48	(8–14)–21	(13–22)–46	9–(23–12)	24–11	(25–10)–45	26–44
^1^Canonical	R–Y	(A–U)–A	(N–N)–R	R–(R–Y)	N–N	(N–N)–N	R–R
Trp	A–C*	(U–U)–A*	(U–A)–A*	A–(A•*A*)	C–G	(C–G)–A	A–A
Lys	A–*A*	(A–A)–A*	(A–U)–A	A–(A–U)	G–C	(C–G)–A	G–A
LeuCUN	G–C*	(A–*A*)–A*	(U–A)–A*	A–(*U*–*A*)	C–G	(C–G)–A	A–G

**Notes:**

Italics letters, nucleotides that do not match with those reported for the same interaction in canonical tRNA.

• Non-canonical pair identified in the 2D structure.

* , Interaction partially supported in the tertiary structure.

1 R, Purines; Y, Pyrimidine; N, any nucleotide.

In tRNA^Lys^, the identity of some bases changed with respect to canonical interactions fundamentally due to the presence of additional mating in the acceptor arm, and in tRNA^Leu (CUN)^, by a larger variable loop. In the tRNA^Trp^ the nucleotide at position 14 intervenes in a tertiary interaction, fact that could explain why the mutation at this position generated changes in the 3D structure that was identified as a different motif. On the other hand, the participation in a tertiary interaction of the non-Watson–Crick A_23_–A_12_ bond identified in the D-arm of the tRNA^Trp^ would indicate why this non-canonical pair is a characteristic conserved in *C. caretta* and in all sea turtles, because the residues involved in tertiary interactions are under a strong selective pressure (Widmann et al., 2010). The bases that form the tertiary interactions in this study (Table 2) are not strictly equivalent to the bases reported by Giegé et al. (2012), nevertheless, the nucleotides that form the tertiary interactions proposed were identified as invariant residues, which provides support, given that the tertiary interactions occur mainly between conserved and semiconserved nucleotides (Widmann et al., 2010).

**Figure 3:**
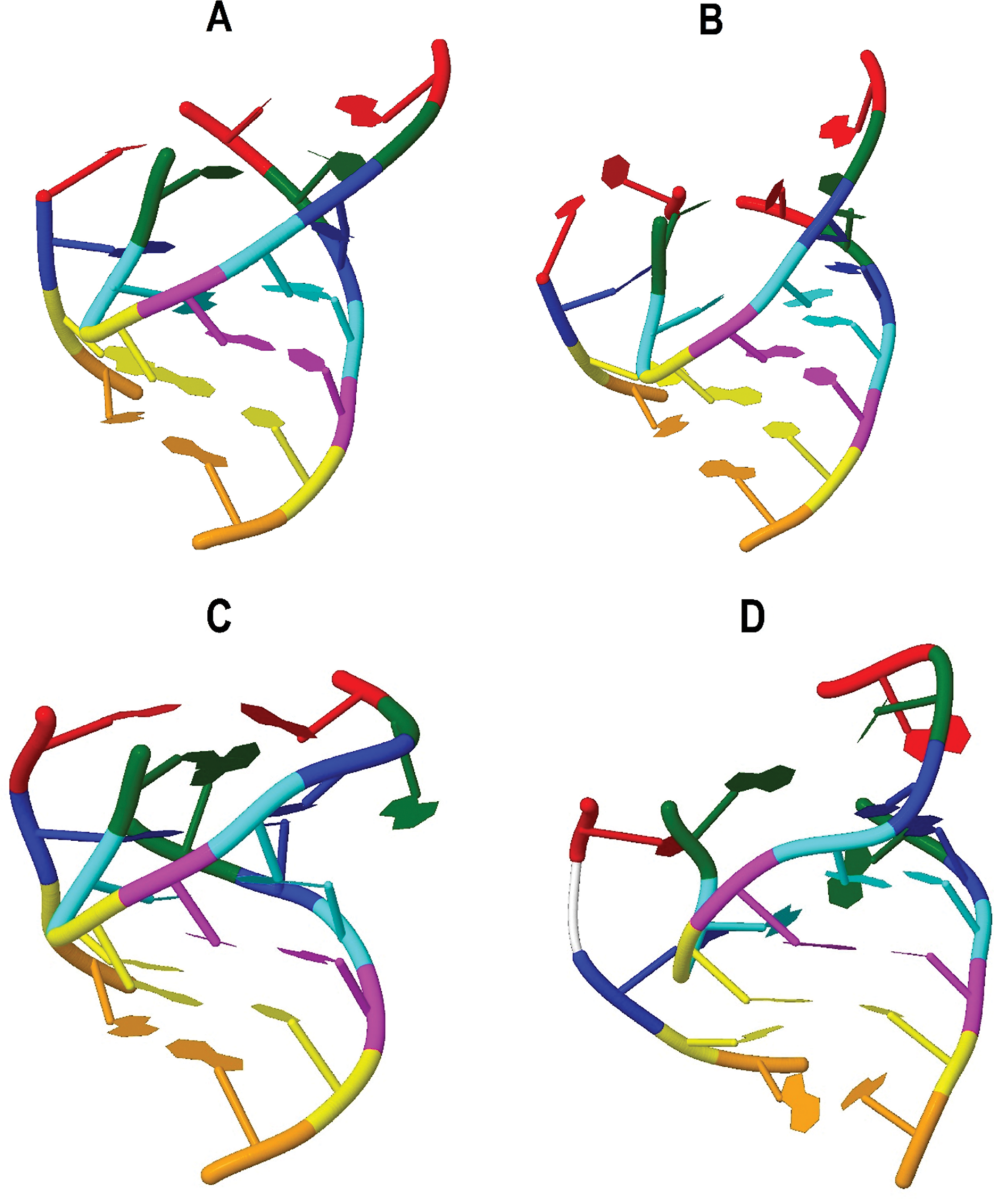
Tertiary interactions identified in putative structures: (A) tRNA^Trp^; (B) tRNA^Trp^ individuals CC5 y CSM-3 (C) tRNA^Lys^; (D) tRNA^Leu(CUN)^. Nucleotides with the same color integrate one interaction as follows: interaction 1-red, 2-green, 3-blue, 4-cyan, 5-magenta, 6-yellow, 7-orange.

Figure 3 shows the verification of the tertiary interactions previously referred. Nucleotides with the same color integrate one interaction and are indicated in Table 2 as follows: interaction 1-red, 2-green, 3-blue, 4-cyan, 5-magenta, 6-yellow, 7-orange. Direct verification on the tertiary structures showed that interactions 1, 2 and 3 are not present in tRNA^Trp^ and tRNA^Leu(CUN)^. Nevertheless, it was observed that interaction 1 in the tRNA^Trp^ (Fig. 3A), known as Levitt pair (Giegé et al., 2012), could be stabilized through the participation of the U_20B_ nucleotide forming the C_48_–U_20B_–A_15_ interaction. In the 3D structure obtained for the CC5 and CSM-3 individuals (Fig. 3B) it was observed that the Levitt pair could be replaced by the U_20_–C_48_ and A_15_–U_20B_ pairs. Interaction 3 in tRNA^Trp^ and tRNA^Leu(CUN)^ (Fig. 3D) does not present pairing of positions 46–22, as described Giegé et al. (2012) for tRNA of class II. In the putative structure of tRNA^Lys^, the invariant base C_48_ was not conserved, nevertheless, the pair A_48_–A_15_ imitates the canonical Levitt pair (Fig. 3C). It looks like interaction 2 is not present in this tRNA.

### Non-Watson–Crick pairs

The comparison of the non-canonical pairs identified in the putative 2D structures obtained in this study with proposed models from studies with X ray crystallography in the RNA of bacteria and yeast (Leontis, Stombaugh & Westhof, 2002) allowed to identify the possible type of non-Watson–Crick pairing, the number of hydrogen bonds that would be forming and the atoms that intervene in such interactions. At the end, it could be determined that all non-canonical pairings may be classified within the same geometrical family, taking into account the border of the nucleotide that participates in the interaction and the orientation of the glycosidic bond according to the axis of interaction given by the hydrogen bonds (Leontis, Stombaugh & Westhof, 2002) within the *Cis Watson–Crick/Watson–Crick family* (Fig. S6). Only pair A_7_–A_66_ was identified as a possible non-complementary pair. According to Leontis, Stombaugh & Westhof (2002), for this case, no example has been identified and there is yet no reasonable model that indicates how the hydrogen bonds would be formed, and thus are classified as non-complementary.

## Discussion

The high percentage of homology identified in the sequences of tRNA^Leu^ and tRNA^Lys^ genes in the seven species of sea turtles show that the genes that are codified as mitochondrial tRNAs are highly conserved (Table 3) (Laslett & Canback, 2008) due to low recombination (Ruiz et al., 2008) and that its primary structure is preserved to maintain discriminating elements and invariant residues that stabilize the secondary and tertiary structure. This is of great importance to fulfill its biological function that depends on defined structural properties (Helm et al., 2000). Still, this contrasts with the high number of polymorphisms identified among the sequences of the tRNA^Trp^ gene in the seven species, evidencing the high speed of mutation and divergence that is present in the mitochondrial DNA (Hebert et al., 2003) which has a a ten-fold mutation rate compared to nuclear DNA (Rubio & Verdecia, 2004). Due to this high variability, mitochondrial DNA is a useful tool for lineage identification (Ruiz et al., 2008). The differences between the sequences were represented mainly by transitions; this could be caused by a deficiency in the repair mechanisms of mitochondrial DNA (Ruiz et al., 2008). The nucleotide composition in the sequences for the three tRNA was dominated by adenine (~38%), cytosine (~27%) and thymine (~20%), only between 15 and 18% corresponded to a guanine for all sequences, as was expected in a mitochondrial gene (Pérez, Bejarano & Vélez, 2008). This fact reflects that tRNA^Trp^, tRNA^Lys^ and tRNA^Leu(CUN)^ are light tRNA, that is, are transcribed from the heavy chain (Helm et al., 2000). The bias in the nucleotide composition is a characteristic of the mitochondrial tRNA of metazoa. It causes the absence of the G18 and G19 bases generating a variation and lack of D/T-loop interactions (Giegé et al., 2012).

**Table 3:** Access numbers to mitochondrial genomes of sea turtles from which the tRNA^Trp^, tRNA^Lys^ and tRNA^Leu(CUN)^ sequences were obtained for multiple alignments.

Species	Access number	Reference
*Dermochelys coriacea*	JX454969.1	Duchene et al. (2012)
	JX454973.1	Duchene et al. (2012)
	JX454989.1	Duchene et al. (2012)
	JX454992.1	Duchene et al. (2012)
*Chelonia mydas*	AB012104	Kumazawa & Nishida (1999)
	JX454974.1	Duchene et al. (2012)
	JX454972.1	Duchene et al. (2012)
	JX454971.1	Duchene et al. (2012)
	JX454976.1	Duchene et al. (2012)
	JX454990.1	Duchene et al. (2012)
*Natator depressus*	JX454975.1	Duchene et al. (2012)
*Eretmochelys imbricata*	NC_012398	Tandon, Trivedi & Kashyap (2006)
	JX454970	Duchene et al. (2012)
	JX454980	Duchene et al. (2012)
	JX454986	Duchene et al. (2012)
*Caretta caretta*	NC_016923.1	Drosopoulou et al. (2012)
	JX454977.1	Duchene et al. (2012)
	JX454983.1	Duchene et al. (2012)
	JX454988.1	Duchene et al. (2012)
	KP256531	Otálora & Hernandez (2015)
*Lepidochelys kempii*	JX454981.1	Duchene et al. (2012)
	JX454982.1	Duchene et al. (2012)
*Lepidochelys olivacea*	JX454979.1	Duchene et al. (2012)
	JX454987.1	Duchene et al. (2012)
	JX454991.1	Duchene et al. (2012)

One constant characteristic in the putative 2D structures of the three tRNA analyzed was the presence of two connector bases between the acceptor stem and the D-stem and one connector base between the D-stem and the anticodon stem; this condition is also described in mammals (Helm et al., 2000). According to the size of the variable region, the three tRNA analyzed belong to class I, because they only contain 4–5 nucleotides (Giegé et al., 2012).

It was observed that critical residues such as the first four base pairs of the acceptor stem, the first three base pairs of the D-stem and the bases that form the anticodon were conserved in each one of the secondary inferred structures. The importance of these nucleotides is that they are discriminatory elements for the recognition of specific aminoacyl-tRNA synthetase, which would explain its conservation given that the nucleotides implied in the interactions with other cell partners are under a strong selective pressure (Widmann et al., 2010). On the other hand, the unpaired base at the 3′-end to which the CCA segment is added, also represents a discriminatory element in some tRNA that ensures the correlation with its specific amino acid (Ibba & Söll, 2000; Alexander, Eargle & Luthey-Schulten, 2010). In the studied tRNAs, this base remained invariant and always corresponded to a purine; adenine in tRNA^Leu^ and tRNA^Lys^ and guanine in tRNA^Trp^, which coincides with Westhof & Auffinger (2001) who describe that this unpaired base is often adenine and less frequently guanine, and few times a pyrimidine.

The interactions of the three tRNA studied (tRNA^Trp^, tRNA^Lys^ and tRNA^Leu(CUN)^) are present mainly between the D-loop and the variable region and between this last one and the space bases which form a compact nucleus (Giegé et al., 2012). This classifies them as type II tRNA (Suzuki, Nagao & Suzuki, 2011) characterized by the lack of canonical D/T-loop interactions. However, the tRNA^Lys^ conserves the G_18_, G_19_, U_55_ and C_56_ bases that could form the interactions between these loops after the postranscriptional modifications. The participation of the connector bases and the variable region in tertiary interactions explains why these residues remained invariant in the secondary structures. Although some interactions are not present in the tertiary motifs, it is possible that the maturity processes could modify the architecture of the tRNA favoring specific interactions (Leontis, Stombaugh & Westhof, 2002; Suzuki, Nagao & Suzuki, 2011).

A global glance shows that in general, the putative structures described in this study for tRNA^Trp^, tRNA^Lys^ and tRNA^Leu^ show motifs that are close to the canonical structures. The secondary structures were placed as a clover leaf reaching a maximum degree of intracatenary pairings (Suzuki, Nagao & Suzuki, 2011) and the tertiary structures matched with the folded “L” archetype, where the arms were placed two by two (T—Acceptor and D—Anticodon) tending towards a coaxial geometry, thus configuring two main adjacent arms that form an angle close to 90° as present in the canonical tRNA (Frazer & Hagerman, 2008). This supports the hypothesis that the 3D structures of the homologous RNA molecules change much slower than its sequences during the course of their evolution (Leontis, Stombaugh & Westhof, 2002). Variations in the lengths of the stems and loops in the 2D structures and distortions or lack of typical interactions in the 3D structures were found, which conferred unique characteristics and which differentiated them between themselves, and with respect to the canonical tRNA. This could be due to the fact that each tRNA molecule has to evolve under two opposing restrictions (Westhof & Auffinger, 2001). On the one hand, it needs a 3D architecture that allows it to fit precisely in the ribosomal binding sites for the promotion of the synthesis of proteins. But on the other hand, it has to have sufficient molecular diversity that will ensure the specific recognition with modifying enzymes, elongation factors and Aminoacyl-tRNA synthase. The fact that they differentiate between them but are adjusted more or less to the same tridimensional size, supposes a notable accomplishment of these molecules that only have an average of 75–85 nucleotides of length (RajBhandary & Soll, 1995). Given the importance of the tRNA structure in the translation of mitochondrial genes, it is plausible that modifications in its structure affect the synthesis of proteins, and therefore, the functional properties of the mitochondria (Calderón, 2014). Nevertheless, the pathogenicity of a mutation depends on factors such as heteroplasmy, the mitotic segregation (Garnacho & Garnacho, 2007) and that it exceeds a threshold, that is, a minimum level of mutated copies so that a phenotypical or biochemical expression of the mitochondria defect exists (Cuadros-Arasa, 2013). The functional studies are essential to fully clarify the mechanisms by which these mutations in the tRNA may result in disease (Blakely et al., 2013). Nevertheless, the comprehension of the numerous functions that RNA performs in living cells critically depends on the knowledge of their tridimensional structure (Popenda et al., 2012) and the comprehension of functional properties of the mitochondrial tRNA lies in the knowledge of its structure (Helm et al., 2000). Therefore, the results presented here, represent a baseline for research for future studies about the functional analysis of the mitochondrial tRNA of *C. caretta* and, in general, sea turtles.

## Conclusions

The structures identified follow the illustrative canonical 3D structures, allowing sufficient conformational flexibility to satisfy the different functional demands of mitochondrial tRNA.

To compensate for the harmful effects of the evolutionary pressure, the tRNA of *C. caretta* could have been deviated from the canonical structures, showing different structural motifs such as the ones presented in this study.

Despite that the molecule databases available online have nucleotide sequences of tRNA of sea turtles, up to this date, no known studies were available regarding the structure of tRNA that operate in the mitochondria of this important taxonomic group in danger of extinction.

The structural and functional knowledge of mitochondrial tRNA acquires notable importance from the perspective of the conservation of a loggerhead turtle if the possible relationship between mutations in these molecules with serious pathologies that threaten the conservation of this species is taken into account.

## Supplemental Information

10.7717/peerj.9204/supp-1 Supplemental Information 1 DNA extraction of loggerhead sea turtles.

10.7717/peerj.9204/supp-2 Supplemental Information 2 PCR Amplification of tRNA genes of loggerhead turtles.

10.7717/peerj.9204/supp-3 Supplemental Information 3 Tryptophan Alignment. tRNA^Trp^ Genes of Loggerhead turtles.

10.7717/peerj.9204/supp-4 Supplemental Information 4 Lysine alignment. tRNALys genes of loggerhead turtlles alignment.

10.7717/peerj.9204/supp-5 Supplemental Information 5 Leucine alignment. tRNA^Leu^ of loggerhead turtles alignment.

10.7717/peerj.9204/supp-6 Supplemental Information 6 W–C Pairs. Watson–Crick links.

## Additional Information and Declarations

### Field Study Permissions

The following information was supplied relating to field study approvals (i.e., approving body and any reference numbers): Field experiments were approved by the Ministry of Environment and Territorial Development (#24 of June 22, 2012).

### Data Availability

The following information was supplied regarding data availability: Data is available at GenBank: KX063643–KX063673.
