# Supplementary figures and images for: Structural analysis of leucine, lysine and tryptophan mitochondrial tRNA of nesting turtles Caretta caretta (Testudines: Chelonioidea) in the Colombian Caribbean
Source: PeerJ. 2020 Jun 18;8:e9204. doi: 10.7717/peerj.9204 (PMC7306221; doi:10.7717/peerj.9204)

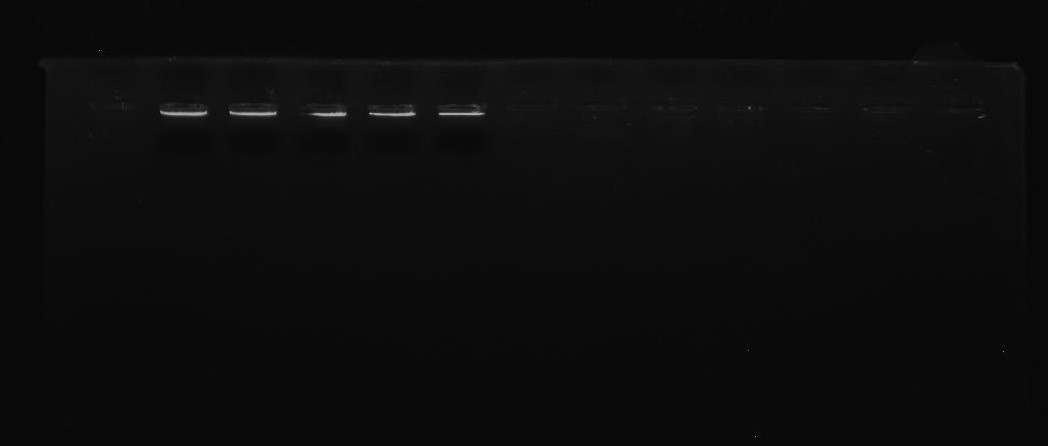

Supplement: Supplemental Information 1 [file peerj-08-9204-s001.png]

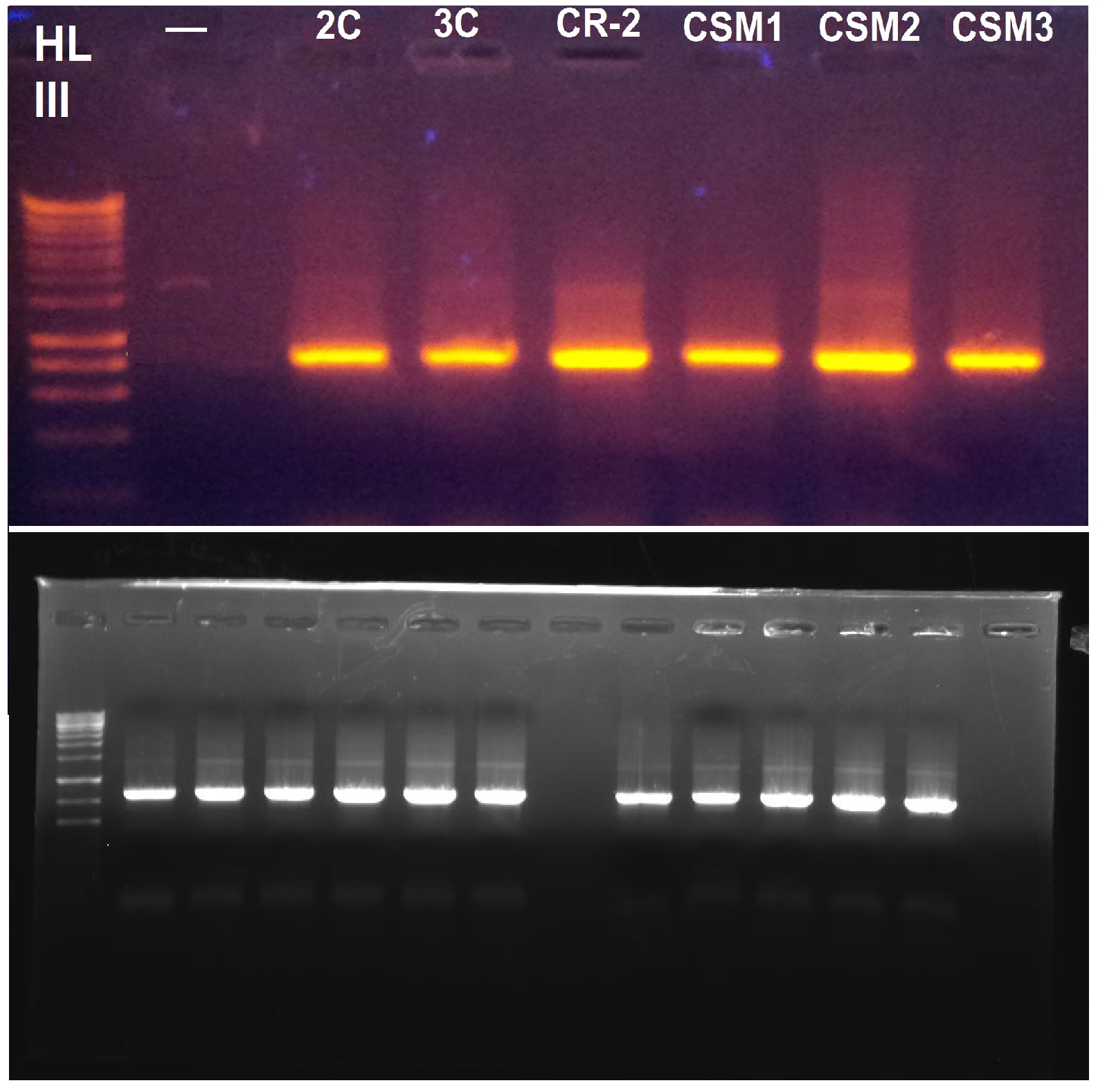

Supplement: Supplemental Information 2 [file peerj-08-9204-s002.png]

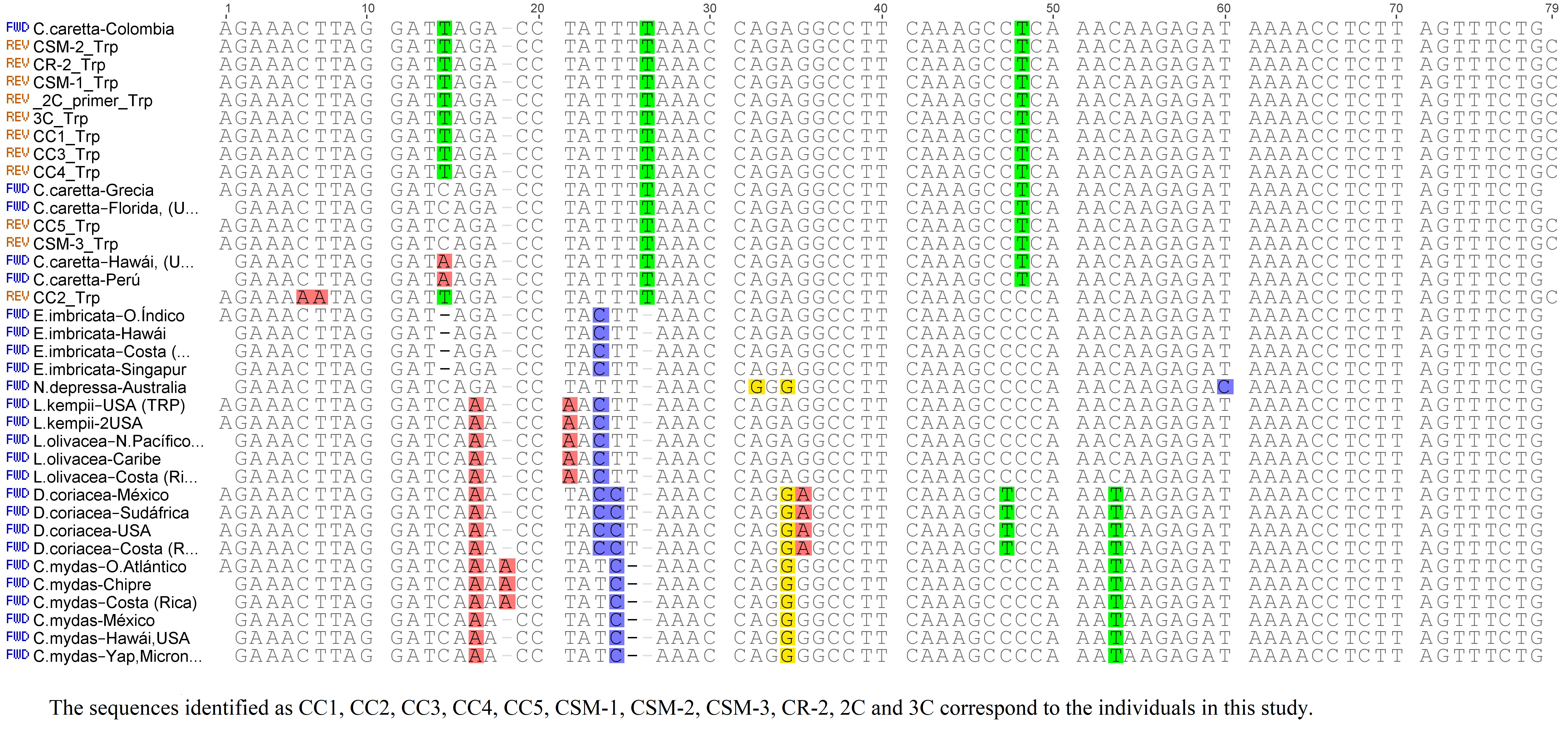

Supplement: Supplemental Information 3 — tRNATrp Genes of Loggerhead turtles. [file peerj-08-9204-s003.png]

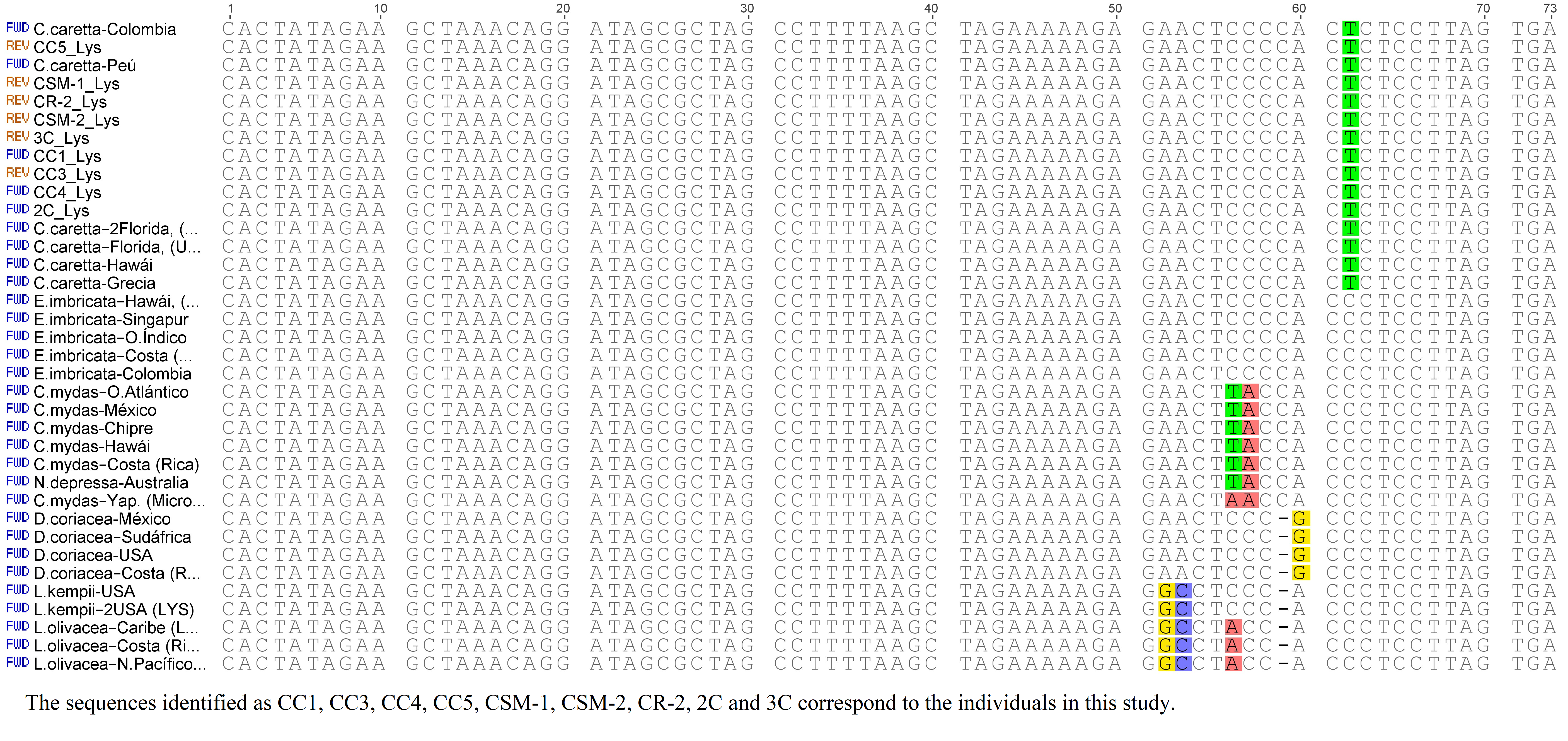

Supplement: Supplemental Information 4 — tRNALys genes of loggerhead turtlles alignment. [file peerj-08-9204-s004.png]

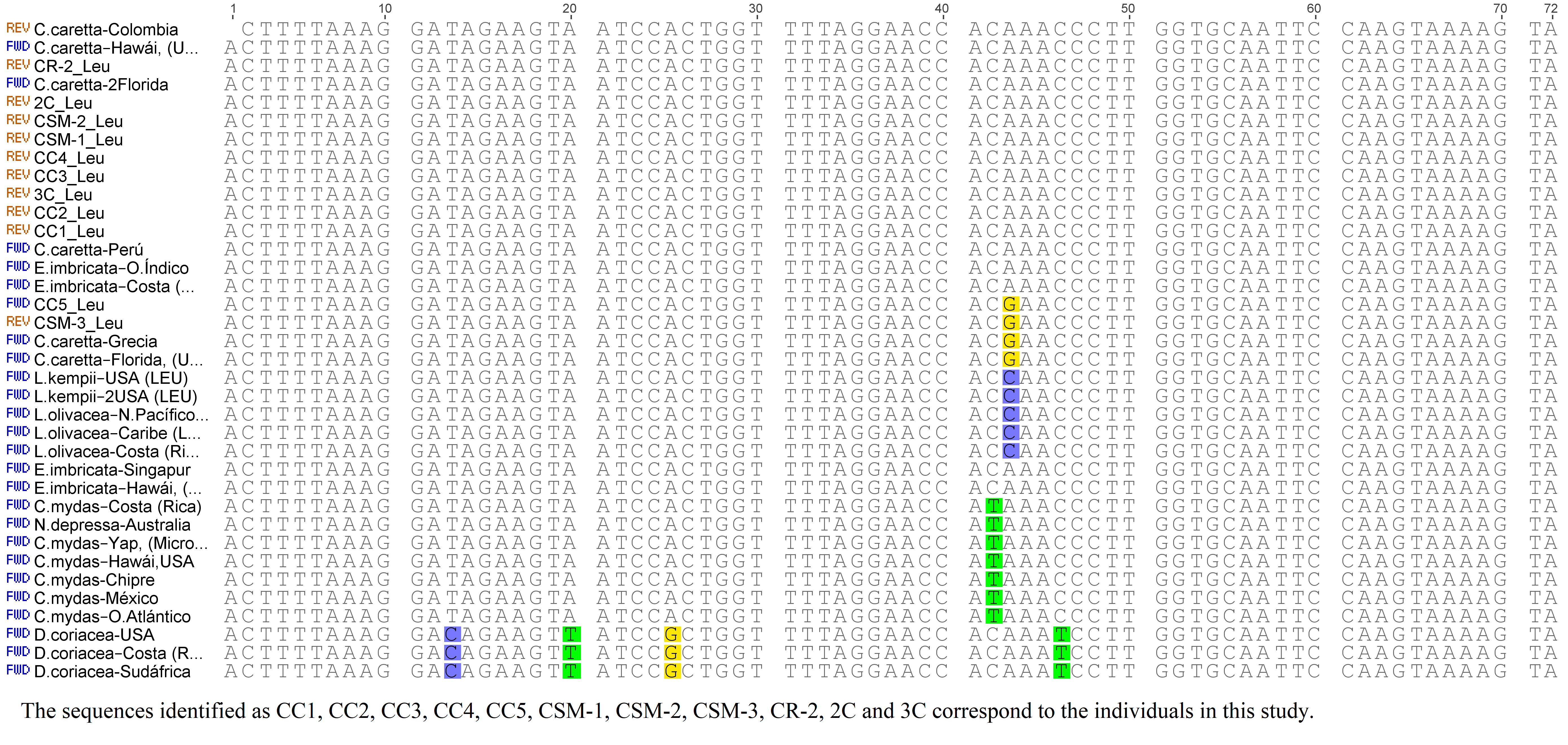

Supplement: Supplemental Information 5 — tRNALeu of loggerhead turtles alignment. [file peerj-08-9204-s005.png]

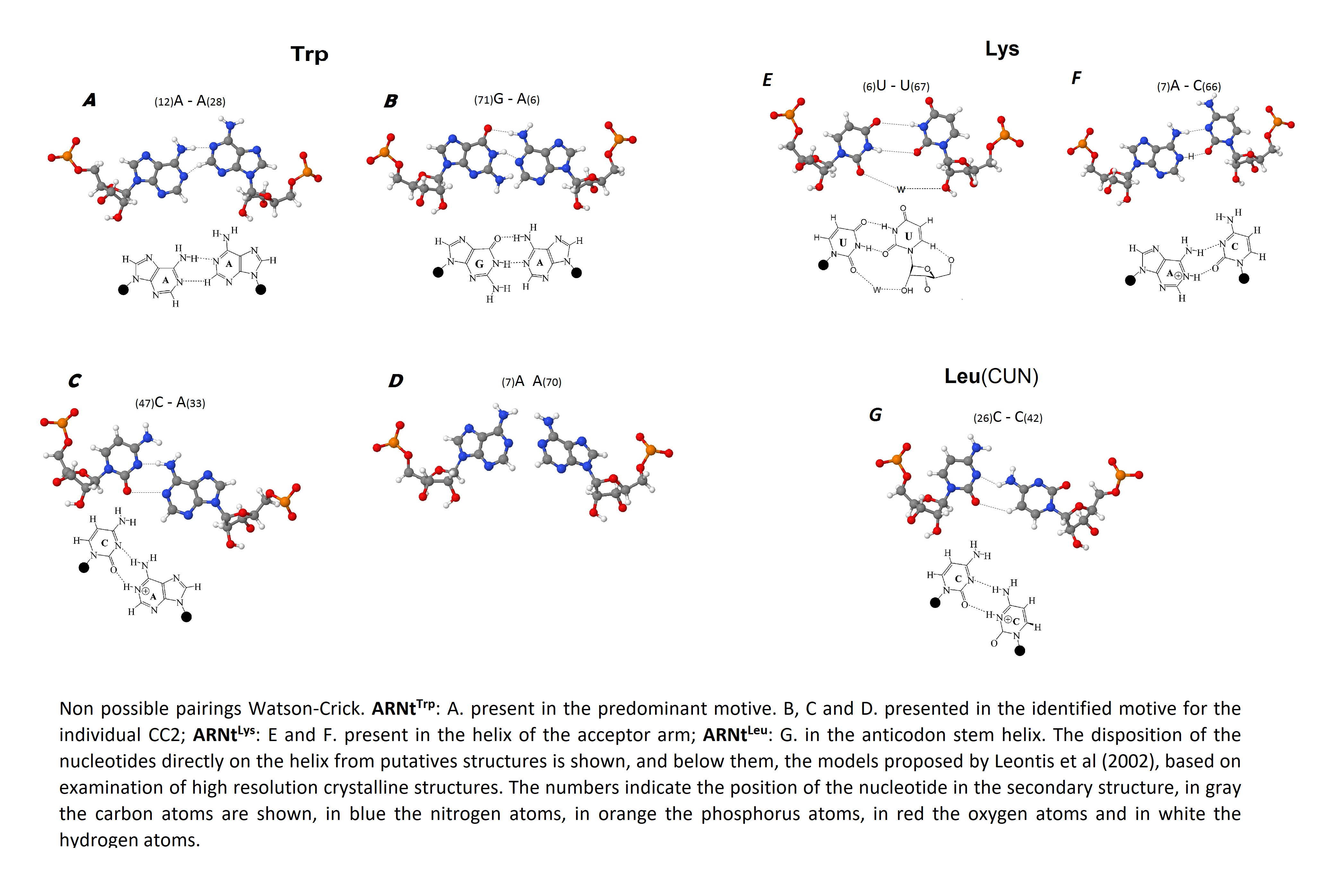

Supplement: Supplemental Information 6 — Watson–Crick links. [file peerj-08-9204-s006.png]
